# Knowledge about childhood autism and opinion among healthcare workers on availability of facilities and law caring for the needs and rights of children with childhood autism and other developmental disorders in Nigeria

**DOI:** 10.1186/1471-2431-9-12

**Published:** 2009-02-12

**Authors:** Muideen O Bakare, Peter O Ebigbo, Ahamefule O Agomoh, Julian Eaton, Gabriel M Onyeama, Kevin O Okonkwo, Jojo U Onwukwe, Monday N Igwe, Andrew O Orovwigho, Chinyere M Aguocha

**Affiliations:** 1 Child and Adolescent Unit, Federal Neuro-Psychiatric Hospital, New Haven, Enugu, Enugu State, Nigeria; 2 Department of Psychological Medicine, University of Nigeria Teaching Hospital, (UNTH), Enugu, Enugu State, Nigeria; 3 General/Forensic Unit, Federal Neuro-Psychiatric Hospital, New Haven, Enugu, Enugu State, Nigeria; 4 West Africa CBM National Co-ordination Office, PO Box 8451, Wuse, Abuja, Nigeria; 5 Community Psychiatry Unit, Federal Neuro-Psychiatric Hospital, New Haven, Enugu, Enugu State, Nigeria

## Abstract

**Background:**

In designing programs to raise the community level of awareness about childhood autism in sub-Saharan Africa, it is logical to use the primary healthcare workers as contact point for education of the general public. Tertiary healthcare workers could play the role of trainers on childhood autism at primary healthcare level. Assessing their baseline knowledge about childhood autism to detect areas of knowledge gap is an essential ingredient in starting off such programs that would be aimed at early diagnosis and interventions. Knowledge of the healthcare workers on availability of facilities and law that would promote the required interventions is also important. This study assessed the baseline knowledge about childhood autism and opinion among Nigerian healthcare workers on availability of facilities and law caring for the needs and rights of children with childhood autism and other developmental disorders.

**Method:**

A total of one hundred and thirty four (134) consented healthcare workers working in tertiary healthcare facilities located in south east and south-south regions of Nigeria were interviewed with Socio-demographic, Knowledge about Childhood Autism among Health Workers (KCAHW) and Opinion on availability of Facilities and Law caring for the needs and rights of children with Childhood Autism and other developmental disorders (OFLCA) questionnaires.

**Results:**

The total mean score of participated healthcare workers on KCAHW questionnaire was 12.35 ± 4.40 out of a total score of 19 possible. Knowledge gap was found to be higher in domain 3 (symptoms of obsessive and repetitive pattern of behavior), followed by domains 1 (symptoms of impairments in social interaction), 4 (type of disorder autism is and associated co-morbidity) and 2 (symptoms of communication impairments) of KCAHW respectively among the healthcare workers. Knowledge about childhood autism (KCA) as measured by scores on KCAHW questionnaire was significantly associated with age group distribution of the healthcare workers, with those age group of fourth decades and above more likely to have higher mean score (p = 0.004) and previous experience of managing children with autism spectrum disorders (ASD) (p = 0.000). KCA showed near significant association with area of specialty, with those healthcare workers in psychiatry compared to pediatrics having higher mean score (p = 0.071) and also with years of working experience of the healthcare workers (p = 0.056). More than half of the healthcare workers subscribed to the opinion that facilities and law caring for the needs and rights of children with childhood autism and other developmental disorders are lacking in Nigeria.

**Conclusion:**

The correlates of KCA may help in selection of those tertiary healthcare workers that would best fit the role of trainers. It is important to update the knowledge gaps of those healthcare workers who scored low in different domains of KCAHW questionnaire. It is imperative for policy makers in Nigeria to advocate and implement multidisciplinary healthcare service system that would ensure early diagnosis and interventions. Nationally representative baseline epidemiological data that would guide policy and planning are also desirable.

## Background

Mode of healthcare delivery system in Nigeria had been in the hierarchy of primary, which provides healthcare for people in the community level, secondary, which provides care for referred cases from primary level and tertiary level which provides specialized care for referred cases from secondary level. Referral pathway is therefore from primary healthcare to secondary healthcare and then to tertiary healthcare centers. There had been repeated call to implement integration of mental healthcare into primary healthcare system in Nigeria [1,2].

Child mental health services have not received adequate attention from policy makers in Nigeria and other sub-Saharan African countries. The need to incorporate child mental health care into primary healthcare system in Africa had been underscored [3]. Information dissemination to promote knowledge and awareness about childhood autism in the community is paramount in Nigeria and there is a great need for policy design and implementation to achieve this objective. Information dissemination about childhood autism could also follow the pathway of healthcare delivery system in the country, whereby healthcare workers working with children at tertiary level of care can play the role of trainers for healthcare workers working with children at secondary and primary level of care in achieving the ultimate objective of promoting knowledge and awareness about childhood autism among these healthcare workers.

Knowledge and awareness about childhood autism is low among healthcare workers working at community level in Nigeria [4,5]. Adequate knowledge and awareness about childhood autism among healthcare workers would ensure early diagnosis of children with autism in the community and this in turn would allow early interventions which had been shown to improve prognosis in children with autism [6-8]. Rhoades et al [9] had earlier noted that healthcare workers' knowledge or lack of knowledge about autism spectrum disorders (ASD) greatly influence the average age of diagnosis and whether the healthcare worker concerned provides further information necessary to care-givers about autism or not, both of which greatly impact on the overall prognosis in children with ASD.

Current literature addressing management of childhood autism and other developmental disorders advocate multidisciplinary approach [10-13], which include involving the children and their parents [14]. This multidisciplinary approach incorporates behavioral therapy, special education, communication and social skill training and management of disruptive behavior with psychotropic medications when indicated [11,12]. Services and professionals that would ensure multidisciplinary approach to management of children with developmental disorders may be lacking in this environment. Also, child right law that protects the interests of children with childhood autism and other developmental disorders is essential to ensuring that the rights of children with developmental disorders in this environment are not compromised as advocated by the 1991 African charter and the 1990 United Nations convention on the rights of the child [15]. The child rights which include the right to health, education, special care among others [16,17] need to be preserved and respected.

This study assessed the baseline knowledge of healthcare workers working with children at tertiary healthcare facilities in south east and south-south regions of Nigeria about childhood autism and determined the correlates. It also assessed the opinion of the same healthcare workers on availability of intervention facilities, training facilities for professionals and law caring for the needs and rights of children with childhood autism and other developmental disorders in Nigeria.

## Methods

### Location

The locations of this study were two tertiary institutions each in south east and south-south regions of Nigeria. One of the two institutions in each region of south east and south-south Nigeria are specialized Psychiatric Hospitals; Federal Neuro-Psychiatric Hospital, Enugu, located in the south-east region of Nigeria and Federal Psychiatric Hospital, Calabar, located in the south-south region of Nigeria. Other two institutions involved in the study were pediatric departments of Ebonyi State University Teaching Hospital (EBSUTH), Abakaliki, located in south east Nigeria and University of Calabar Teaching Hospital (UCTH), Calabar, located in south-south region of Nigeria.

### Ethical approval

The ethical approval for the study was obtained from the Institutional Review Board (IRB) of Federal Neuro-Psychiatric Hospital, New Haven, Enugu, Enugu State, Nigeria.

### Participants and sampling method

The healthcare workers involved in the study were nurses, mostly with diploma qualification in nursing either working at the two specialized psychiatric hospitals in south east and south-south regions of Nigeria or working in the department of pediatrics of the two tertiary teaching hospitals located in the south east and south-south regions of the country. Therefore, the nurses involved in the study were either working in the area of specialty of psychiatry or pediatrics and had been working in one of these areas of specialty in a tertiary institution for at least one year. The study was a point survey of knowledge of the healthcare workers about childhood autism. A point sampling method that involved all nurses on their duty posts in the four different institutions on the particular day the data were collected was employed. All nurses on their duty posts in the four different institutions for that particular day gave their consent and were interviewed.

## Materials

### Socio-demographic questionnaire

This was used to obtain demographic information like gender, age, marital status, area of specialty, years of working experience, previous involvement with managing of children with autism spectrum disorders (ASD) among others.

### Knowledge about childhood autism among health workers (KCAHW) questionnaire (Appendices 1 and 2)

Knowledge about childhood autism among health workers (KCAHW) questionnaire is a nineteen item questionnaire that is divided into four domains. The questionnaire scores knowledge of healthcare workers about childhood autism in the four domains. Detail description and scoring of the questionnaire can be found in our earlier study [5] and its content and scoring method had also been reproduced in appendices 1 and 2 respectively. KCAHW questionnaire was used to assess the baseline knowledge about childhood autism among the healthcare workers that participated in this study.

### Opinion on availability of facilities and law caring for the needs and rights of children with childhood autism and other developmental disorders (OFLCA) questionnaire (Appendix 3)

This questionnaire was designed to obtain information from the healthcare workers about their opinion on availability of intervention facilities for children with childhood autism and other developmental disorders in Nigeria. Also, about their opinion on availability of training facilities for all professionals that are likely to be involved in the multidisciplinary approach to management of childhood autism and other developmental disorders in Nigeria. The questionnaire also assessed the opinion of the healthcare workers on availability of law that protect the rights and interests of children with childhood autism and other developmental disorders in Nigeria. The OFLCA questionnaire is shown in Appendix 3.

### Procedure

The socio-demographic questionnaire, KCAHW and OFLCA questionnaires were administered to the healthcare workers that consented to participate in the study. The questionnaire were given to the participants to complete there and then and the questionnaires were collected back immediately to avoid consultation of study materials in responding to the questions on KCAHW questionnaire since the questionnaire was meant for a point time assessment of knowledge [5].

### Data analysis

The data were analyzed using Statistical Package for Social Sciences (SPSS), version 15. The mean, median and mode scores of the total score for the healthcare workers on KCAHW questionnaire were calculated. The various mean scores in relation to the socio-demographic variables of the healthcare workers were also compared using one way Analysis of Variance (ANOVA) to determine the correlates of knowledge scores on KCAHW questionnaire. Frequency and percentage distribution of the healthcare workers' opinions were also computed.

## Results

A total of one hundred and thirty four (134) healthcare workers consented to participate in the study. There were 71 (53.0%) males and 63 (47.0%) females. The mean age of the participants is 35.89 ± 7.56 years. Other socio-demographic variables are shown in Table 1.

**Table 1:** Socio-demographic variables of the healthcare workers

**Socio-demographic Variables**	**N (%)**
**Age Group (Years)**	
20 – 29	28 (20.9)
30 – 39	57 (42.5)
40 – 49	44 (32.8)
50 and Above	5 (3.7)
	
**Gender**	
Male	71 (53.0)
Female	63 (47.0)
	
**Marital Status**	
Single	38 (28.4)
Married	91 (67.9)
Separated/Divorced	1 (0.7)
Widowed	4 (3.0)
	
**Area of Specialty**	
Pediatrics	21 (15.7)
Psychiatry	113 (84.3)
	
**Working Experience (Years)**	
1 – 5	61 (45.5)
6 – 10	9 (6.7)
11 – 15	16 (11.9)
16 – 20	41 (30.6)
20 and Above	7 (5.2)
	
**Geographical Region**	
South East	62 (46.3)
South-South	72 (53.7)
	
**Previous Involvement with Management of Children with Autism Spectrum Disorders (ASD)**	
Previous Involvement	65 (48.5)
No Previous Involvement	69 (51.5)

### Pattern of distribution of scores on KCAHW questionnaire among the healthcare workers

Knowledge about childhood autism among health workers (KCAHW) questionnaire allowed a minimum score of zero (0) and a maximum score of nineteen (19) and it is divided into four domains with maximum total scores of 8, 1, 4 and 6 possible in domains 1, 2, 3 and 4 respectively of the questionnaire and a minimum score of 0 is possible in each of the four domains [5]. The total mean score on KCAHW questionnaire among the healthcare workers that participated in the study was 12.35 ± 4.40. The median score was 13.00 and the mode score was 15.00.

The mean score in domain 1, which deals with questions on the area of impairments in social interaction found in childhood autism was 5.80 ± 1.90 and a total of 80 (59.7%) of the healthcare workers scored within and above the mean score in this domain. The mean score in domain 2 which deals with question on communication impairments that often characterized childhood autism was 0.82 ± 0.14 and a total of 107 (79.9%) of the healthcare workers scored within and above the mean score in this domain. Domain 3, which deals with questions on obsessive and repetitive behavioral pattern that often characterized childhood autism had a mean score of 2.20 ± 0.90 and a total of 54 (40.3%) of the healthcare workers scored within and above the mean score in this domain. Domain 4 that addressed questions on what type of disorder is childhood autism and possible associated co-morbidity recorded a mean score of 3.53 ± 1.46 and a total of 80 (59.7%) of the healthcare workers scored within and above the mean score in this domain. A total of 94 (70.2%) of the healthcare workers scored within and above the total mean score of 12.35 ± 4.40 in the four domains. Knowledge gap was found to be higher in domain 3, followed by domains 1, 4 and 2 respectively of KCAHW questionnaire.

The pattern of distribution of scores in the different domains of KCAHW questionnaire is shown in Table 2.

**Table 2:** The pattern of distribution of scores in the different domains of KCAHW questionnaire among the healthcare workers

**Domains**	**Area of Knowledge/Symptoms Questions Addressed**	**Total Score Possible**	**Mean Scores**	**Number of Healthcare Workers with Scores within and above the Mean Scores** N (%)
**Domain 1**	Impairments in Social Interaction	8	5.80 ± 1.90	80 (59.7)

**Domain 2**	Impairment in Communication	1	0.82 ± 0.14	107 (79.9)

**Domain 3**	Obsessive and Repetitive Behavioral Pattern	4	2.20 ± 0.90	54 (40.3)

**Domain 4**	Type of Disorder Autism is and Possible Associated Co-morbidity	6	3.53 ± 1.46	80 (59.7)

**Summation of Domains 1, 2, 3 and 4**	Summation of Scores in the four domains	19	12.35 ± 4.4	94 (70.2)

**Figure 1:**
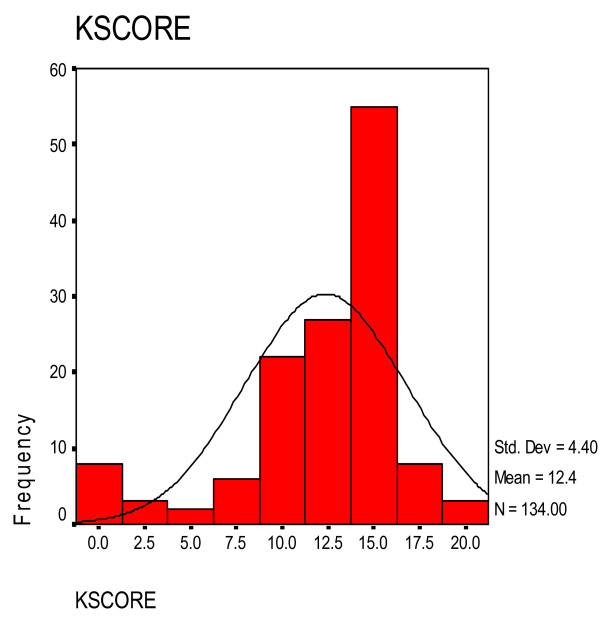
**Histogram and Normal Distribution Curve of Health Care Workers' Scores on KCAHW Questionnaire**. KSCORE – Scores on KCAHW Questionnaire. Frequency is plotted on the Y-Axis, while KCAHW Questionnaire scores are plotted on X-Axis.

The histogram and normal distribution curve showing pattern of scores from minimum of 0 to maximum of 19 on KCAHW questionnaire among the healthcare workers is shown in Figure 1.

### Correlates of knowledge about childhood autism among the healthcare workers

Significant relationship was found between mean score on KCAHW questionnaire and age group distribution of the healthcare workers. Significant relationship was also found between mean score on KCAHW questionnaire and experience of past involvement with managing children with autism spectrum disorders (ASD).

Healthcare workers in their fourth decade of life and above tend to have higher mean score on KCAHW questionnaire (F-Ratio = 4.6, df = 3, p-value = 0.004). Healthcare workers who have had the previous experience of being involved in management of children with ASD were more likely to have higher mean score on KCAHW questionnaire when compared to those who have not had such experience before (F-Ratio = 30.5, df = 1, p-value = 0.000).

Near significant relationship was found between mean score on KCAHW questionnaire and area of specialty or practice of the healthcare workers. Near significant relationship was also found between mean score on KCAHW questionnaire and years of working experience of the healthcare workers. Healthcare workers working in tertiary psychiatric facilities were more likely to have higher mean score on KCAHW questionnaire when compared to those working in department of pediatrics of tertiary healthcare facilities

(F-Ratio = 3.3, df = 1, p-value = 0.071). Healthcare workers at the extremes of years of working experience, those with less than six years working experience and those with more than twenty years of working experience were more likely to have a low mean score on KCAHW questionnaire when compared to those healthcare workers whose working experience were between six and twenty years (F-Ratio = 2.4, df = 4, p-value = 0.056). Comparison of mean scores on KCAHW questionnaire in relation with various socio-demographic variables of the healthcare workers is shown in Table 3.

**Table 3:** Comparison of mean scores on KCAHW questionnaire in relation to the healthcare workers' socio-demographic variables

**Socio-demographic Variables**	**Mean Scores on KCAHW Questionnaire**	**One way ANOVA Comparing Mean Scores**
**Age Groups (Years)**		
20 – 29	9.8 ± 5.2	
30 – 39	13.4 ± 3.4	F-Ratio = 4.6, df = 3,
40 – 49	12.5 ± 4.6	p-value = 0.004*
50 and Above	13.6 ± 2.3	

**Gender**		
Male	12.9 ± 4.0	F-Ratio = 2.3, df = 1,
Female	11.8 ± 4.8	p-value = 0.134

**Marital Status**		
Single	11.8 ± 4.0	
Married	12.4 ± 4.6	F-Ratio = 1.5, df = 3,
Separated/Divorced	15.0 ± 0.0	p-value = 0.215
Widowed	16.5 ± 2.4	

**Area of Specialty**		
Pediatrics	10.8 ± 4.4	F-Ratio = 3.3, df = 1,
Psychiatry	12.7 ± 4.4	p-value = 0.071^#^

**Working Experience (Years)**		
1 – 5	11.8 ± 4.4	
6 – 10	13.6 ± 2.1	F-Ratio = 2.4, df = 4,
11 – 15	15.2 ± 2.4	p-value = 0.056^#^
16 – 20	12.0 ± 5.1	
21 and Above	11.0 ± 3.7	

**Geographical Region**		
South East	12.3 ± 4.2	F-Ratio = 0.01, df = 1,
South-South	12.4 ± 4.6	p-value = 0.977

**Previous Involvement with Management of Children with Autism Spectrum Disorders (ASD)**		
Have been Involved	14.3 ± 2.1	F-Ratio = 30.5, df = 1,
Never been Involved	10.5 ± 5.1	p-value = 0.000*

* Significant relationship between mean scores on KCAHW questionnaire and the healthcare workers' socio-demographic variables.

^# ^Near significant relationship between mean scores on KCAHW questionnaire and the healthcare workers' socio-demographic variables.

### Opinion of the healthcare workers on availability of intervention facilities for childhood autism and other developmental disorders

Forty five (33.6%) of the healthcare workers had the opinion that there are intervention facilities that meet both the medical and social needs of children with childhood autism and other developmental disorders in Nigeria, these healthcare workers often specify psychiatric facilities in their geographical location as examples of those facilities, while 89 (66.4%) had the opinion that such intervention facilities are unavailable. None (0%) of the healthcare workers was unsure about availability of such intervention facilities in Nigeria.

### Opinion of the healthcare workers on availability of training facilities for all the professionals who are likely to be involved in the multidisciplinary approach of management of childhood autism and other developmental disorders

Twenty one (15.7%) of the healthcare workers had the opinion that training facilities for all the professionals who are likely to be involved in the multidisciplinary approach to management of childhood autism and other developmental disorders are available in Nigeria, these healthcare workers often specify schools of psychiatric nursing and residency training programs for pediatricians and psychiatrists as examples of such training facilities, while 112 (83.6%) of the healthcare workers thought such training facilities to train all these professionals are not available in Nigeria. One (0.7%) of the healthcare workers was unsure whether such training facilities for all these professionals are available in Nigeria.

### Opinion of the healthcare workers on availability of law that protects the rights and interests of children with childhood autism and other developmental disorders

Seven (5.2%) of the healthcare workers expressed the opinion that there is law that protect the rights and interests of children with childhood autism and other developmental disorders, these healthcare workers specified child right law as such law, while 71 (53.0%) expressed the opinion that such law is not in existence in Nigeria. Fifty six (41.8%) of the healthcare workers were unsure whether such law is in existence in Nigeria or not.

Table 4 showed the frequency and percentage distributions of healthcare workers' opinions on availability of intervention facilities, training facilities for professionals and law caring for the needs and rights of children with childhood autism and other developmental disorders in Nigeria. Significantly more healthcare workers were likely to subscribe to the opinion that intervention facilities, training facilities for professionals and law caring for the needs and rights of children with childhood autism and other developmental disorders are lacking in Nigeria.

**Table 4:** Frequency and percentage distributions of healthcare workers' opinions on availability of intervention facilities, training facilities for professionals and law caring for the needs and rights of children with childhood autism and other developmental disorders in Nigeria

**Opinion of the Healthcare Workers**	**Intervention Facilities** **N (%)**	**Training Facilities** **N (%)**	**Law** **N (%)**
**Available**	45 (33.6%)	21 (15.7%)	7 (5.2%)
**Unavailable**	89 (66.4%)	112 (83.6%)	71 (53.0%)
**Unsure**	0 (0%)	1 (0.7%)	56 (41.8%)

**Total**	134 (100%)	134 (100%)	134 (100%)

## Discussion

### Knowledge about childhood autism

The mean score on KCAHW questionnaire among the healthcare workers studied was 12.35 ± 4.40. This revealed a much higher level of knowledge about childhood autism compared to the mean scores of 9.6 and 9.8 obtained among the healthcare workers manning the community mental health outposts in our earlier study [5]. The possible explanation would be that these groups of healthcare workers working at tertiary level of healthcare facilities are more equipped with relatively more knowledge about childhood autism, which would aid early diagnosis and identification of cases. Knowledge gap was found to be higher in domain 3, followed by domains 1, 4 and 2 respectively of KCAHW questionnaire among the healthcare workers. With further training to fill up the identified areas of knowledge gap, they may be able to assume the role of trainers for other healthcare workers working at secondary and primary level of healthcare system in this environment.

Knowledge about childhood autism among the healthcare workers was found to be significantly related to age group distribution of the healthcare workers. Healthcare workers in their fourth decade of life and above tend to show more knowledge about childhood autism compared to those who are younger. This finding may be a reflection of working experience in their various specialty of practice. Those healthcare workers who had previous experience of being involved in management of children with ASD were also more likely to show more knowledge about childhood autism as reflected by their mean score on KCAHW questionnaire. This is further reinforcing the important roles of working experience and previous involvement with care in baseline knowledge about childhood autism.

Near significant relationship was also found between areas of specialty of the healthcare workers and knowledge about childhood autism. Those healthcare workers working primarily in psychiatric facilities tend to show more knowledge about childhood autism compared to those working in pediatrics departments of the tertiary healthcare facilities studied. Though, management of children with ASD requires multi-disciplinary approach, the children with ASD in this environment are more likely to be brought to psychiatric facilities for attention because of the associated behavioral problems, possible associated epilepsy and mental retardation which most lay people in Nigeria would think are in the area of practice of psychiatrists, only second to traditional or other unorthodox care center.

Near significant association was also found between knowledge about childhood autism and years of working experience of the healthcare workers. Those healthcare workers with less than six years working experience and those with more than twenty years of working experience were likely to have lower mean scores on KCAHW questionnaire compared to those with working experience of between six and twenty years. The possibility that this finding could be due to the relatively recent upsurge in the awareness about childhood about a decade and half ago in this environment was entertained. This may make the healthcare workers with few years of working experience and those with longer years of working experience not to be that familiar with cases of ASD. This further pose a necessity of advocating incorporation of recent research findings on ASD into teaching curriculum of nurses and other healthcare workers in this environment, this may improve the knowledge of younger healthcare workers with limited number of years of experience in practice.

The discrepancy in knowledge found among the healthcare workers working in specialties of pediatrics and psychiatry in our study is consistent with findings of previous surveys of knowledge about childhood autism among healthcare workers that had documented some differences and misconceptions about various aspects of childhood autism across disciplines [18-20].

### Opinion on availability of facilities and law caring for the needs and rights of children with developmental disorders

Significantly more healthcare workers, more than half of the participated healthcare workers had the opinion that intervention facilities which meet the medico-social needs of children with childhood autism and other developmental disorders are not available in Nigeria. More healthcare workers also had the opinion that training facilities for training all professionals that would likely be involved in the multidisciplinary approach to management of children with childhood autism and other developmental disorders are lacking in Nigeria. Very few healthcare workers, about five percent subscribed to the opinion that there is child right law in Nigeria that addresses the needs and rights of children with childhood autism and other developmental disorders.

The above findings revealed the approximate true position on the issues of facilities, but contrary to the true position on presence or availability of child right law in Nigeria. The knowledge of these healthcare workers may need to be updated in this regard.

### Childhood autism and community mental healthcare in Nigeria

The importance of improving and updating the mental health knowledge of community mental healthcare workers by continuous training had been identified as; improved diagnostic acumen of specific mental health disorders in the community and improved ability to provide helpful adequate and necessary information to the patients and their care-givers regarding the management [2], and also a change of attitude and perception that reduce stigmatization and discrimination [21,22].

Community mental healthcare in Nigeria and other parts of the world often employed the mixture of consultation and linkage model, of which continued education of community mental healthcare workers constitutes an integral part. In designing policy to promote knowledge and awareness about childhood autism in Nigeria and thereby ensuring early diagnosis and identification of cases, the tertiary healthcare workers who are suppose to work out the consultation and linkage modalities at primary care level have a significant role to play. If they are to play the significant role in dissemination of information and providing training to the community healthcare workers about childhood autism, the need to assess their own baseline knowledge as done by this study, to detect and subsequently fill the knowledge gaps in various areas of deficiencies can not be overemphasized.

In their report of World Health Organization (WHO) collaborative study on strategies for extending mental healthcare, Murthy and Wig [23] described the training approach aimed at enhancing the availability of mental health man power in developing world to include; evaluation of workers' existing knowledge of and attitudes towards mental disorders, evaluation of existing training materials, evaluation of the need for new training materials and evaluation of the support and supervision needed to carry out their duties. It is our suggestion that any policy and research aimed at promoting knowledge and awareness and management of childhood autism in various communities in Nigeria should follow this same approach. Therefore, future research in this environment would need to approach other relevant areas of need in training of healthcare workers in the community about childhood autism and improving the general knowledge and awareness at community level. This would help to alleviate the plight of children with childhood autism and other developmental disorders and also ease the burden of care among their parents.

### The present position on availability of facilities and law caring for children with childhood autism and other developmental disorders in Nigeria

The present situation is that there are some intervention facilities to meet the needs of children with childhood autism and other developmental disorders in Nigeria. These intervention facilities however fall short of expectation of meeting most medico-social needs of children with developmental disorders. Those healthcare workers that had the opinion that intervention facilities that meet the medico-social needs of children with childhood autism and other developmental disorders are available in Nigeria were likely to cite government owned psychiatric facilities in their environs as the perceived intervention facilities. However, these psychiatric facilities in Nigeria are not well equipped to meet the multidisciplinary approach to management of childhood autism and other developmental disorders. Other facilities that could address the areas of management of childhood autism and other developmental disorders related to special education, communication and social skill training are often lacking. While the medical needs of physical co-morbidity and behavioral problems associated with childhood autism and other developmental disorders could be met by the existing facilities in Nigeria, the parents of these children often express great unmet needs in the area of education and other social needs.

Professionals like child psychiatrists, pedagogist, speech-language pathologists and special educators that can contribute to the multidisciplinary approach of managing childhood autism and other developmental disorders are often grossly lacking. This raised the issue of training and availability of training facilities for these professionals in this environment.

Studies coming from developed parts of the world had documented dissatisfaction among the families of children with developmental disabilities with their primary healthcare physicians because of unmet needs in healthcare delivery system, which was more notable among parents of children with childhood autism [24]. Such dissatisfaction and unmet needs with primary healthcare delivery system could stem from limited knowledge of healthcare workers on needs of children with autism and other developmental disorders [9] or non availability of professional expertise to provide the holistic approach to management that is required in children with developmental disorders.

Such scenarios of unmet needs among parents of children with developmental disorders are also prevalent in Nigeria and other sub-Saharan African regions. Unmet needs in healthcare delivery system to children with developmental disabilities and their parents can constitute extra burden of care and psychological distress to the parents [25] and it had been suggested that addressing such unmet needs in children with developmental disability may reduce psychological complications in the parents [25].

The intricacy of socio-cultural, political and economic factors contributing to inadequate healthcare facilities and training facilities for healthcare workers and allied professionals to adequately meet the medico-social needs of children with developmental disorders and child mental health services in general in Nigeria had earlier been discussed by Olatawura [26].

Child right law takes care of the rights and privileges of children in general and also of children with special needs and disabilities under which children with childhood autism and other developmental disorders are classified. Nigeria had been a signatory to the United Nations Convention on the Rights of the Child since 1991 and the African Charter on the Rights and welfare of the Child since 2001 [27]. Nigeria adopted the Child Right Act into law in 2003 and since then effective implementation and execution had been clogged by ignorance, peculiar socio-cultural and political factors [27].

The small proportion of about five percent of the participated healthcare workers that had knowledge of existence of child right law compare to the largest percentage that had the opinion that child right law is not in existence in Nigeria or were unsure is a reflection of the imperative need to educate the healthcare workers and other stakeholders on availability of such law that protects the interests of children with special needs. Further education of children, parents, teachers, healthcare workers and law enforcement agents about the child right law in Nigeria may go a long way in ensuring effective implementation and execution of the law.

## Conclusion

Healthcare workers working in tertiary healthcare facilities that showed good knowledge about childhood autism may be able to adopt the role of trainers for those healthcare workers working at primary healthcare or community level, who in turn would be the contact point for the education of general public in the communities about childhood autism. While the associated correlates of knowledge about childhood autism may help in selection of those tertiary healthcare workers that would best fit this role, it is also important to update the knowledge gaps of other tertiary healthcare workers who scored low on different domains of KCAHW questionnaire. This would help to improve early diagnosis of cases and interventions which influence the prognosis [6-8].

There is great need for special pediatric healthcare delivery system in this environment which must be multidisciplinary in nature to address the present areas of unmet needs in children with childhood autism and other developmental disorders and also the needs of their parents. Before this can be achieved however, baseline epidemiological data that would guide policies and planning are needed. The prevalence and distributions of children with developmental disorders in this environment need to be ascertained. Evaluation of intervention and training facilities presently in existence needs to be done.

Unmet needs of children with childhood autism and other developmental disorders in the present healthcare delivery system need to be assessed.

Future studies aiming at providing baseline data to guide policies and planning on healthcare delivery system to children with childhood autism and other developmental disorders in Nigeria should focus on these issues.

## Competing interests

The authors declare that they have no competing interests.

## Authors' contributions

All authors contributed to the conception of the study. MOB wrote the initial draft of the manuscript. MOB, POE, AOA, JE, GMO were involved in revising of the manuscript. All authors read and approved the final draft of the manuscript.

## Appendix 1

### Knowledge about Childhood Autism among Health Workers (KCAHW) Questionnaire

**Please do not consult formal text books to answer these questions**.

**Thank you for your time**.

The following behaviors best describe a child with Childhood Autism:

#### Domain 1

i. Marked impairment in use of multiple non-verbal behaviors such as eye to eye contact, facial expression, body postures and gestures during social interaction?

(A) Don't Know, (B) Yes, (C) No

ii. Failure to develop peer relationship appropriate for developmental age?

(A) Don't Know, (B) Yes, (C) No

iii. Lack of spontaneous will to share enjoyment, interest or activities with other people? (A) Don't Know, (B) Yes, (C) No

iv. Lack of social or emotional reciprocity? (A) Don't Know, (B) Yes, (C) No

v. Staring into open space and not focusing on any thing specific?

(A) Don't Know, (B) Yes, (C) No

vi. The child can appear as if deaf or dumb? (A) Don't Know, (B) Yes, (C) No

vii. Loss of interest in the environment and surroundings?

(A) Don't Know, (B) Yes, (C) No

viii. Social smile is usually absent in a child with Autism?

(A) Don't Know, (B) Yes (C) No

#### Domain 2

i. Delay or total lack of development of spoken language?

(A) Don't Know (B) Yes (C) No

#### Domain 3

i. Stereotyped and repetitive movement (e.g. Hand or finger flapping or twisting)?

(A) Don't Know (B) Yes, (C) No

ii. May be associated with abnormal eating habit?

(A) Don't Know, (B) Yes, (C) No

iii. Persistent preoccupation with parts of objects?

(A) Don't Know, (B) Yes, (C) No

iv. Love for regimented routine activities? (A) Don't Know, (B) Yes, (C) No

#### Domain 4

i. Autism is Childhood Schizophrenia? (A) Don't Know, (B) Yes, (C) No

ii. Autism is an auto-immune condition? (A) Don't Know, (B) Yes, (C) No

iii. Autism is a neuro-developmental disorder? (A) Don't Know, (B) Yes, (C) No

iv. Autism could be associated with Mental Retardation?

(A) Don't Know, (B) Yes, (C) No

v. Autism could be associated with Epilepsy? (A) Don't Know, (B) Yes, (C) No

vi. Onset of Autism is usually in, (A) Neonatal age, (B) Infancy, (C) Childhood

## Appendix 2

### Scoring of Knowledge about Childhood Autism among Health Workers (KCAHW) Questionnaire

#### Domain 1

i Marked impairment in use of multiple non-verbal behaviors such as eye to eye contact, facial expression, body postures and gestures during social interaction?

(A) 0 (B) 1 (C) 0

ii Failure to develop peer relationship appropriate for developmental age?

(A) 0 (B) 1 (C) 0

iii. Lack of spontaneous will to share enjoyment, interest or activities with other people? (A) 0 (B) 1 (C) 0

iv Lack of social or emotional reciprocity? (A) 0 (B) 1 (C) 0

v Starring into open space and not focusing on any thing specific?

(A) 0 (B) 1 (C) 0

vi. The child can appear as if deaf or dumb? (A) 0 (B) 1 (C) 0

vii. Loss of interest in the environment and surroundings?

(A) 0 (B) 1 (C) 0

viii. Social smile is usually absent in a child with Autism?

(A) 0 (B) 1 (C) 0

#### Domain 2

i. Delay or total lack of development of spoken language?

(A) 0 (B) 1 (C) 0

#### Domain 3

i. Stereotyped and repetitive movement (e.g. Hand or finger flapping or twisting)?

(A) 0 (B) 1 (C) 0

ii. May be associated with abnormal eating habit?

(A) 0 (B) 1 (C) 0

iii. Persistent preoccupation with parts of objects?

(A) 0 (B) 1 (C) 0

iv. Love for regimented routine activities? (A) 0 (B) 1 (C) 0

#### Domain 4

i. Autism is Childhood Schizophrenia? (A) 0 (B) 0 (C) 1

ii. Autism is an auto-immune condition? (A) 0 (B) 0 (C) 1

iii Autism is a neuro-developmental disorder? (A) 0 (B) 1 (C) 0

iv. Autism could be associated with Mental Retardation?

(A) 0 (B) 1 (C) 0

v. Autism could be associated with Epilepsy? (A) 0 (B) 1 (C) 0

vi Onset of Autism is usually in (A) 0 (B) 0 (C) 1

**A total maximum score of 19 and a minimum score of 0 are possible. The average score on the KCAHW questionnaire among a particular sample population give an index level of knowledge about childhood autism in that particular population**.

## Appendix 3

### Opinion on Availability of Facilities and Law caring for the needs and rights of Children with Childhood Autism and other Developmental Disorders (OFLCA) Questionnaire

Kindly answer the following questions to the best of your knowledge and opinion: Make a choice and give explanation for your choice. Thank you for your time.

1. Are there healthcare institutions or facilities in Nigeria that provide multidisciplinary care and support to meet the medico-social needs of children with childhood autism and other developmental disorders?

(A) YES (B) NO

Please give explanation for your choice and examples if necessary ______________________________________________________________________________________

2. Are there training facilities in Nigeria that provide training for all the professionals who are likely to be involved in the multidisciplinary approach to management of children with childhood autism and other developmental disorders?

(A) YES (B) NO

Please give explanation for your choice and examples if necessary________________________________________________________________________________________

3. Is there any law or regulation in Nigeria that protects the rights and interests of children with childhood autism and other developmental disorders?

(A) YES (B) NO

Please give explanation for your choice and examples if necessary__________________________________________________________________________________________

## Pre-publication history

The pre-publication history for this paper can be accessed here:


